# Anterior Cortical Impingement With 240 mm Cephalo-Medullary Nail (PFN and PFNA-2) in Intertrochanteric Femur Fracture Fixation in the Indian Population: A Problem Statement

**DOI:** 10.7759/cureus.68207

**Published:** 2024-08-30

**Authors:** Devashis Barick, Ameya Sawarkar, Suhas Waghe, Pranav Suradkar, Akhilesh S Khobragade, Virendra E Patil

**Affiliations:** 1 Orthopaedics and Traumatology, N. K. P. Salve Institute of Medical Sciences (NKPSIMS) and Research Centre (RC) Lata Mangeshkar Hospital, Nagpur, IND; 2 Orthopaedics and Trauma, N. K. P. Salve Institute of Medical Sciences (NKPSIMS) and Research Centre (RC) Lata Mangeshkar Hospital, Nagpur, IND; 3 Orthopaedics, N. K. P. Salve Institute of Medical Sciences (NKPSIMS) and Research Centre (RC) Lata Mangeshkar Hospital, Nagpur, IND

**Keywords:** garden alignment index, proximal femoral nail, proximal femoral nail antirotation (pfna), inter-trochanteric fracture, anterior cortical impingment

## Abstract

Trochanteric fractures are common in the elderly population, and their incidence increases twice every decade after age 50. Intramural fixation has achieved good clinical efficacy in the treatment of unstable trochanteric fractures, but there have been complications reported in the literature in the Asian population. Most complications arise from a mismatch between the increase in the anterior femoral bow with advancing age and the proximal femoral nails (PFN) on the market, which still have straight designs on the sagittal plane. The non-anatomic shapes of the PFNs sometimes make the surgeries difficult or may lead to an inadvertent intraoperative fracture around the tip of the nail, particularly if they impinge on the anterior cortex of the femur.

The entry point on the greater trochanter was divided into three equal parts, i.e., anterior 1/3rd, middle 1/3rd, and posterior 1/3rd on the lateral X-rays. Patients with posterior 1/3rd entry were excluded from the study as it is known that posterior positioning of nail entry can cause an increased incidence of anterior nail impingement. The AI was measured using the best available preoperative lateral roentgenogram of the femur using the incidence cortex (AI cortex) angle. This angle was measured using two tangential lines drawn parallel to the anterior cortex of the femur, proximal and distal to the most bowed point of the femur.

We recommend that there is a need to introduce anterior curvature in the sagittal plane corresponding to the femoral bow in a 240 mm cephalomedullary nail to decrease complications. We also consider the use of either a short (i.e., 180 mm) or a long cephalomedullary nail in the Indian population, as the height of the population is shorter as compared to the western population, and the role of a 240 mm cephalomedullary nail is doubtful in the Indian population.

## Introduction

Elderly people frequently suffer from trochanteric fractures, which become more common beyond the age of 50 and increase in frequency twice a decade [1]. Stable fixation is essential for safe and prompt mobilization following trochanteric fractures (TF), and it is critical that patients resume their pre-fracture activity level [2]. As the mechanical axis of the body and the intramedullary system is parallel, it is superior to the extramedullary system. It has an extra biomedical advantage in treating unstable trochanteric fractures compared to extramedullary devices. The nonanatomic forms of proximal femoral nails (PFNs) can occasionally complicate surgery or increase the risk of an unintentional intraoperative fracture around the nail tip, especially if they impinge on the femur's anterior cortex. The increased femoral bending may further increase the strains at this location [3]. Patients who are shorter and have shorter femurs are more likely to have this issue, particularly those who are Caucasian and Asian [1]. Our goal is to investigate the prevalence of anterior cortical impingement in the intertrochanteric femur fracture fixation in the Indian population using 240 mm cephalo-medullary nails (PFN and PFNA-2 [proximal femoral nail antirotation-2]).

## Materials and methods

Written informed consent was obtained from patients to be included in the study. All the data was filled up in case record form and compiled in Microsoft Excel 2017 (Microsoft® Corp., Redmond, WA) to calculate mean, average, and standard deviations wherever necessary.

For sample size estimation

As per calculation

p (proportion of cortical impingement) = 38

q = 100 - p

l(error) = + 10

sample size = 4pq/l^2^; therefore, the required sample size is 94.

The data include age, sex, height, BMI, fracture classification, quality of fracture reduction on Garden Alignment Index, angle of incidence cortex, angle of incidence of canal, entry point on greater trochanter, and position of nail tip in the canal. Patients with intertrochanteric femur fractures were screened based on inclusion and exclusion criteria. X-rays of the afflicted hip with thigh AP and hip with thigh lateral were used for the first examination in patients who were selected. Follow-up X-rays were performed on a one-month interval to measure the radiological index, which includes the entry site on the Greater Trochanter, the angle of canal incidence, and the location of the nail tip. On lateral X-rays, the greater trochanter entry site was separated into three equal sections, or the anterior, middle, and posterior 1/3rd. Since posterior nail entry placement is known to enhance the frequency of anterior nail impingement, patients with posterior 1/3rd entry were excluded from the investigation. The study only examined the front and middle 1/3rd Greater Trochanter entries. The lateral X-ray will show the nail tip's location, categorized as anterior, neutral, or posterior.

The anterior nail tip position was further divided into five grades of impingement. Grade 1: anterior internal cortex contact is absent, but it is situated anteriorly; Grade 2: contact between the nail and internal cortex but less than 33% of the anterior cortical thickness; Grade 3: modest abutment. Impingement is moderate, with more than 33% but less than 76% of the anterior cortex thickness; Grade 4: more than 76% of the thickness of the anterior cortex, severe abutment; Grade 5: anterior cortex perforation.

**Figure 1:**
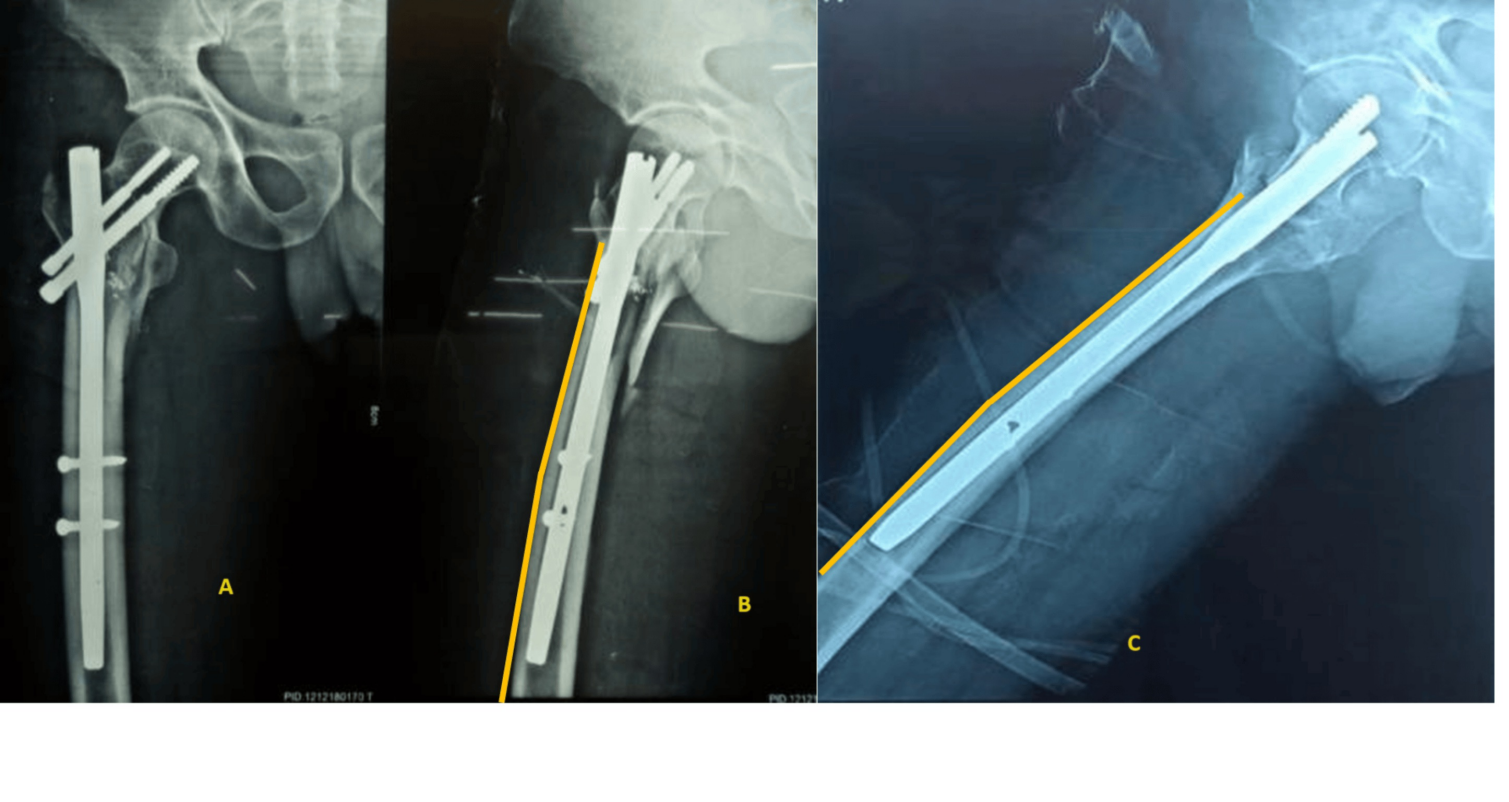
X-ray of hip with proximal femur showing anterior cortex (AI cortex) (A) Antero posterior view; (B) and (C) lateral view; yellow line: tangential line parallel to anterior cortex of femur proximal and distal to the most bowed point of the femur

Both the anterior cortex and the preoperative lateral X-ray were utilized. AI is calculated by two parallel lines from the anterior cortex of the femur, proximal and distal-most bent points, and used to measure angle (Figure 1).

## Results

**Figure 2:**
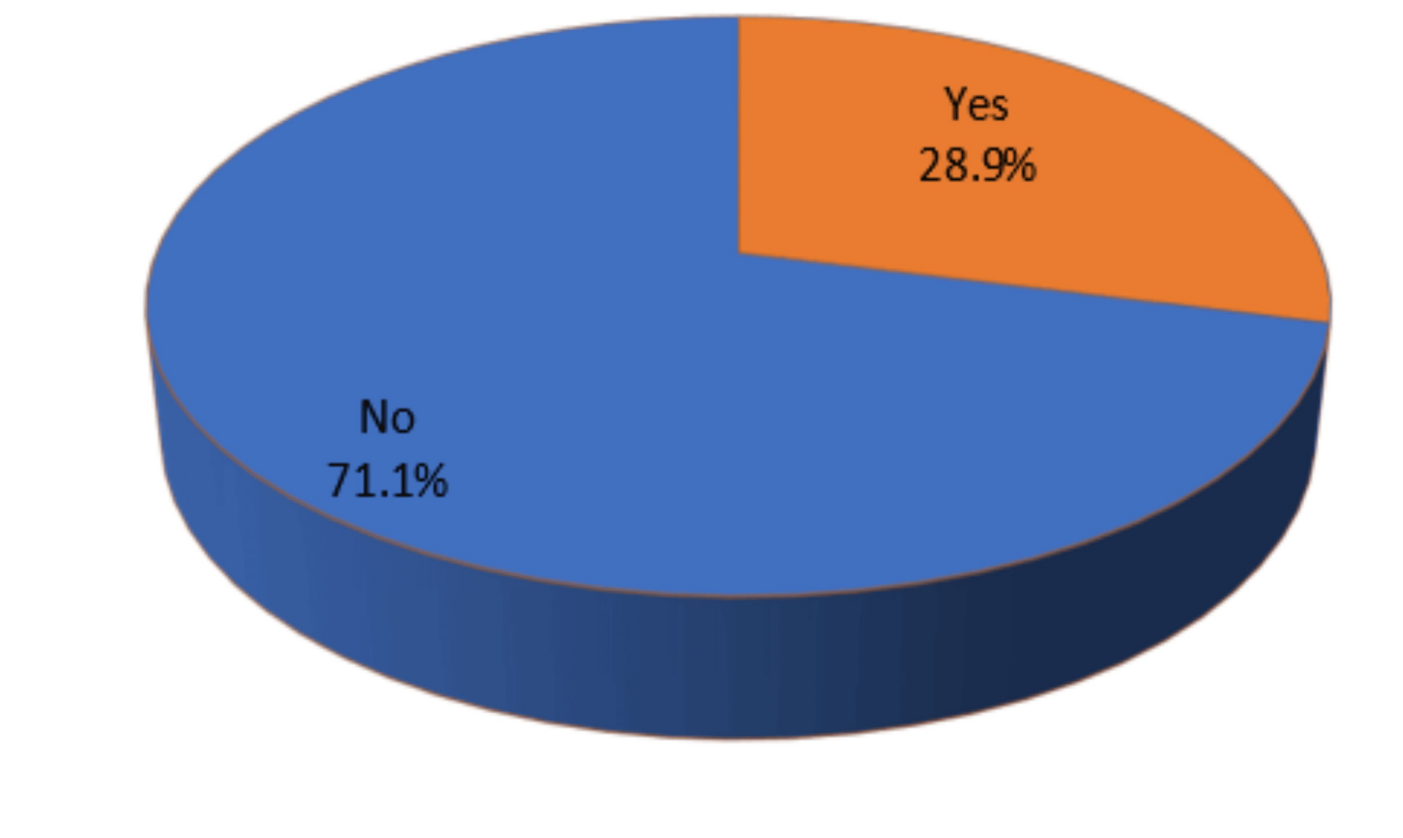
Pie chart showing incidence % of anterior cortical impingement N-value = 187; yellow = cortical impingement; blue = no cortical impingement

We included 187 patients treated with a 240 mm cephalomedullary nail for intertrochanteric fracture, of which 92 were male and 95 were female, corresponding to 49.2% of males and 50.8% of the females in the study (Figure 2). The average age was 66.9 years with 13.8 standard deviation, which corresponds to a minimum to maximum between 39 and 105 years, and the mean height was 158.1 cm with a standard deviation of 4.9, which ranges between 150 and 170 cm, and the weight was 55.3 kg with a standard deviation of 5.0, which ranges between 44 and 68 kg, and BMI 22.1 with a standard deviation of 1.6, which ranges between 19 and 28 kg/m^2^ (Table 1). Sixty-nine patients had no cortical impingement, defined as a nail located anteriorly but not in contact with the anterior internal cortex; 64 patients had slight abutment, defined as nail contact but less than 1/3 of the anterior cortex thickness; 16 patients had moderate abutment, defined as a nail with more than 1/3 but less than 2/3 of the anterior cortex thickness; and 38 patients had severe abutment, defined as more than 2/3 of the anterior cortex thickness (Table 2). Out of a total N value of 187, Group 1 had no cortical impingement (no impingement and slight abutment), and Group 2 had moderate and severe abutments. One hundred and thirty-three patients had no impingement, which corresponds to 71.1%, and 54 patients had significant impingement, which corresponds to 28.9%, with a total incidence of 28.9% and a 95% confidence interval for an incidence rate between 22.5% and 35.9% (Table 3). We also correlated the angle of incidence of cortex with age with Pearson’s correlation coefficient and significance. P-value with interpretation for linear correlation of age with r = (0.5678) and P = 0.001 is strongly positive and significant; for height, r = (−0.5781) and P = 0.001 is strongly negative and significant, and for weight, r = (−0.4443) and P = 0.001 is fairly strongly negative and significant; for BMI, r = (−0.0462) and P = 0.5301 is non-significant and non-correlation (Table 4). The angle of incidence of the cortex was higher in older and shorter patients. Out of the total of 44 thigh pain patients, 38 are actually having anterior cortical impingement, and five are not having cortical impingement; out of 143 non-thigh pain patients, 15 are having cortical impingement but still do not have thigh pain, and the rest 128 patients do not have cortical impingement, hence not having thigh pain (Table 5).

**Table 1:** Demographics of study subjects (n=187) SD: standard deviation

Characteristics	Number	%
Male/female	92 /95	49.2%/50.8%
	Mean ± SD	Min–Max
Age (years)	66.9 ± 13.8	39–105
Height (cm)	158.1 ± 4.9	150–170
Weight (kg)	55.3 ± 5.0	44–68
BMI (kg/m^2^)	22.1 ± 1.6	19–28

**Table 2:** Grade of anterior cortical impingement

Grade	Number	%
Located anteriorly but no contact with the anterior internal cortex	69	36.9
Contact but less than 1/3 of the anterior cortex thickness, slight abutment between nail and internal cortex	64	34.2
More than 1/3 but less than 2/3 of the anterior cortex thickness, moderate abutment	16	8.6
More than 2/3 of the anterior cortex thickness, severe abutment	38	20.3
Total	187	100

**Table 3:** Incidence of anterior cortical impingement N-value = 187; CI: confidence interval

Impingement	Number
Yes	54 (28.88%)
No	133 (71.12%)
Total	187
Incidence (%)	28.88%
95% CI for incidence rate	22.5–35.9%

**Table 4:** Correlation of angle of incidence of cortex with demographics of study subjects (n=187) n = sample size (187); p < 0.001: highly significant; r = Pearson’s correlation coefficient

Angle of incidence of cortex correlated with	r (Pearson’s correlation coefficient)	Significance (P-value)	Interpretation for linear correlation
Age (years)	0.5678	0.001*	Strong, positive, significant
Height (cm)	−0.5781	0.001*	Strong, negative, significant
Weight (kg)	−0.4443	0.001*	Fairly strong, negative, significant
BMI	−0.0462	05301 (NS)	Non-significant and no correlation

**Table 5:** Association of anterior cortical impingement with anterior thigh pain Pearson chi-square (df=1) = 100.05; P < 0.001: highly significant; risk (RR) = 8.5; 95%CI (5.2–13.8)

Impingement	Thigh pain number (%)	No thigh pain number (%)
Yes	39 (88.6)	15 (10.5)
No	5 (11.4)	128 (89.5)
Total	44 (100.0)	143 (100.0)

## Discussion

Since the femoral bow is intact in intertrochanteric fractures, as opposed to subtrochanteric, midshaft, or distal end fractures, where mismatch is corrected by angulation at the fracture site or over-reaming, anterior cortical penetration is a severe issue in PFN or PFNA treatment [4,5]. The femur's anterior curvature influences the ease of inserting an intramedullary nail. Even with a light nail hammer, this might result in problems such as shaft femur fracture or cortical penetration, particularly in elderly patients with osteoporosis. Age has a substantial correlation with both cortical and medullary bowing because older people-especially women-have larger anterior bows than younger people do [3]. Black people have a larger femur curvature radius than white people [6]. This may require modification of orthopedic implants based on racial differences.

In the coronal plane, the PFN/PFNA that AO introduced had a medial-lateral angle of 6°. The nail is straight in the sagittal plane and does not adjust for the femur's natural anteversion. Because of its design, it may be utilized on either side, which helps to lower the implant's arsenal and expense. One of the most popular hip nail systems worldwide is now the PFN/PFNA. However, there have not been many reports of technical challenges caused by the mismatch between the medullary canal and the PFN or PFNA (Synthes, Pennsylvania, PA, USA). [3]

In 1998, the AO developed the PFN to treat intertrochanteric fractures. In 2003, it was changed to PFNA due to difficulties related to PFN. The femoral geometry of Americans and Europeans served as the model for this implant's development. It is well known that Asian and Western populations differ in the geometry of the femur. A newly designed PFNA-II for the Asian population was modified from the first version of PFN after Leung et al. [7,8] reported issues related to geometric mismatch of gamma nails and Chinese femurs.

Three modifications were made to accommodate Asian anatomical features: A flat proximal lateral surface so that it will prevent impingement with the femoral lateral cortex, reduced mediolateral angle in the coronal plane from 6 to 5 degrees, and shortened proximal segment diameter from 17 to 16.5 mm [5]. Nonetheless, in the sagittal plane, all of PFN A2's short nails (180 and 240 mm) are straight [4,5]. 

There have been many studies showing cortical impingement with PFN nailing in the Chinese population [4,5, 8-12], but no studies have been conducted in the Indian population with the standard 240 mm PFN. However, there have been reported complications due to mismatch in many studies [13-15]. Our study emphasizes anterior cortical impingement due to the standard length of PFN, i.e., 240 mm cephalomedullary nail in the Indian population. In this study, we found that 54 patients out of 187 patients had moderate and severe abutment of the nail to the anterior cortex. However, we had no patients with cortical perforation. The incidence of cortical impingement was 28.9%.

PFNA (Synthes, Solathum, Switzerland) has a well-established history of causing valgus impingement of the lateral cortex or femur fractures, particularly in Asian patients [16]. These results from the literature have led to revisions and improvements in its proximal diameter, lateral bending angle, and lateral surface, which are now recreated as PFNA-II, which is demonstrated to be more suitable for the Asian population. Because of its low rate of malunion and rotational strength, InterTan PFN has been shown to be superior in biomechanical and clinical investigations pertaining to the treatment of unstable TFs. In addition to increasing the risk of problems, including thigh discomfort and incapacity, anterior cortical impingement or femoral nail destruction can also act as a stress raiser for subsequent fractures [2].

Anterior thigh pain and the possibility of periprosthetic fractures are frequently linked to cortical impingement happening anteriorly. The distal tip of a small cephalomedullary nail is intended to be positioned before the apex of the femoral bow. On the other hand, the distal end of the nail is positioned near the peak of the femoral curve in the Indian population because of their shorter stature and greater femoral bow curvature. Forty-four participants in our study experienced anterior cortical impingement-related thigh pain.

Due to anterior cortical impingement, the nail gets jammed distally. Due to jamming, there is no role in dynamic locking distally, as no compression occurs at the fracture site. This can also predispose to non-union and implant failure. This type of failure has been reported by Maniscalco et al. [17], in which later on total hip arthroplasty was performed. However, no such case occurred in our study. But the incidence of a nail getting jammed distally is more in 240 mm of cephalomedullary nail. Hwang et al. [5] recommended the use of long cephalomedullary nails in such instances of jamming.

The limitation of this study is that it was conducted in our institute for research purposes only, which included a small study group and was restricted to a limited area. In our suggestion, a similar multicentric study can be conducted so that the overall Indian population is included and a larger sample size can be obtained to validate the obtained conclusion.

## Conclusions

In our study, we have observed that the femoral bow is higher in short-height and old-age populations. In the Indian population, due to short height and advancing age, the femoral bow is higher as compared to the Western population, due to which the incidence of anterior cortical impingement with a 240 mm cephalomedullary nail is higher. It has also been observed that anterior cortical impingement is significantly associated with anterior thigh pain. Moreover, there is also an increased risk of anterior cortical fracture near the tip of the nail during nail insertion. In order to overcome these complications, we recommend that there is a need to introduce anterior curvature in the saggital plane corresponding to the femoral bow in a 240 mm cephalomedullary nail to decrease above said complications. We also consider the use of short and long cephalomedullary nails in the Indian population, as the height of the population is shorter as compared to the western population, and the role of a 240 mm cephalomedullary nail is doubtful in the Indian population.
